# Dr. Boring

**Published:** 1859-02

**Authors:** 


### DR. BORING.

We sincerely regret that this excellent Physician and able Divine, has consented once more to leave his native State, where he has been so long, and so favorably known to both of the professions with which he has been associated, to enter upon a distant field of ministerial labor. At the recent Session of the Georgia Conference, held in Columbus, he was at his request, transferred to the Rio Grand Conference, Texas, and stationed at the city of San Antonio, where his amiable character and high pulpit abilities, cannot fail to secure for him, from an appreciating public, that respect and admiration which they are so well calculated to inspire.

A pleasant and intimate association, for three successive years with Dr. Boring, while occupying the Chair of Obstetrics in the Atlanta Medical College, fully warrants this voluntary expression of our high respect for the talents of the Professor, and the worth of the man.

As a *teacher* of Medicine, Dr. B. had few equals—perhaps no superior in the United States, especially in that department assigned him in the College. Gifted with a high order of intellect—cultivated by close application to his favorite themes, and with quick and clear perception, and a discriminating judgment—any subject instantly assumed form and system at the touch of his plastic mind. Nor was this all. His fine powers of elocution—his deliberate, clear, and forcible enunciation, together with his rare capability of simplifying the most perplexing and abstruse subjects for the easy comprehension of the youngest and most untrained of his hearers—gave him a commanding control over his attentive and delighted Classes.

His uniform and consistent piety, his ample Theological resources, and his urbane and dignified deportment, in connection with his rhetorical claims, and his clear and convincing logic, must ever secure for him a high position in the ministry of that church which he has so long, and so sacrificingly served.

Dr. Boring carries with him to his new and distant home, the warm and affectionate regards of his old confreres of the Atlanta Medical College, who *unite* in this testimonial to his worth, and in the expression of their sincere aspirations for the happiness of himself and family, for *both worlds.*

